# What Influences Educators’ Design Preferences for Bullying Prevention Programs? Multi-level Latent Class Analysis of a Discrete Choice Experiment

**DOI:** 10.1007/s12310-019-09334-0

**Published:** 2019-06-22

**Authors:** Charles E. Cunningham, Heather Rimas, Tracy Vaillancourt, Bailey Stewart, Ken Deal, Lesley Cunningham, Thuva Vanniyasingam, Eric Duku, Don H. Buchanan, Lehana Thabane

**Affiliations:** 1 Department of Psychiatry and Behavioural Neurosciences, Faculty of Health Sciences, McMaster University, 1280 Main St W, Hamilton, ON L8S 4L8 Canada; 2 Counselling Psychology, Faculty of Education, University of Ottawa, 145 Jean-Jacques-Lussier, Ottawa, ON K1N 6N5 Canada; 3 DeGroote School of Business, McMaster University, 1280 Main St W, Hamilton, ON L8S 4L8 Canada; 4 Hamilton-Wentworth District School Board, 20 Education Court, Hamilton, ON L9A 0B9 Canada; 5 Department of Health Research Methods, Evidence, and Impact, Faculty of Health Sciences, McMaster University, 1280 Main St W, Hamilton, ON L8S 4L8 Canada; 6 Offord Centre for Child Studies, McMaster Innovation Park, Suite 201A, 175 Longwood Rd. S, Hamilton, ON L8P 0A1 Canada; 7 Ron Joyce Children’s Health Centre, 237 Barton Street East, Hamilton, ON L8L 2X2 USA

**Keywords:** Bullying, Educator preferences, Discrete choice experiments, Psychological Reactance Theory

## Abstract

We used a discrete choice conjoint experiment to model the anti-bullying (AB) program preferences of 1080 junior kindergarten to Grade 8 educators. Participants chose between hypothetical AB programs that varied combinations of 12 design attributes. Multi-level latent class analysis yielded three classes: *All*-*in Supervisors* (21.5%) preferred that all teaching staff supervise playgrounds and hallways; *Facilitators* (61.6%) preferred that students take ownership of AB activities with 25% of educators supervising playgrounds and hallways; and *Reluctant Delegators* (16.9%) preferred delegating the supervision of playgrounds and hallways to non-teaching staff. This class reported higher dispositional reactance, more implementation barriers, and more psychological reactance to these initiatives. They were less sensitive to social influences and less intent on participating in AB activities. Multi-level analysis showed a greater proportion of *Reluctant Delegators* clustered in one of the two groups of schools. The program choices of all classes were sensitive to the support of principals, colleagues, students, and, to a lesser extent, parents. All classes preferred programs conducted from kindergarten through Grade 12 that addressed the problems underlying bullying while valuing firm and consistent consequences for all students. Educators preferred AB programs selected by individual schools, rather than governments.

**Electronic supplementary material:**

The online version of this article (10.1007/s12310-019-09334-0) contains supplementary material, which is available to authorized users.

## Introduction

Bullying represents the repeated, intentional targeting of students by more powerful peers (Olweus, 1994). Between 10 and 33% of students report involvement in bullying as a target with 5% to 13% acknowledging involvement as perpetrators (Hymel & Swearer, 2015). Victimization of children and youth by peers is associated with academic difficulties, rejection by peers, low self-esteem, anxiety, and depression (McDougall & Vaillancourt, 2015). Prospective longitudinal studies show that children and youth victimized by their peers are at increased risk of adult mental health problems (McDougall & Vaillancourt, 2015). Although anti-bullying (AB) programs yield modest reductions in bullying during the elementary school years (Ttofi & Farrington, 2011; Yeager, Fong, Lee, & Espelage, 2015), within-study analyses suggest that the impact of AB programs beyond middle school is more limited (Yeager et al., 2015).

Fidelity is critical to the implementation and outcome of AB initiatives. In a study of 7413 students, preparation for and adherence to the Finnish KiVa AB program’s protocols were associated with class level decreases in student-reported victimization (Haataja et al., 2014). In a US longitudinal study, dosage, a latent variable composed of the percentage of the KiVa program’s activities educators completed, the number of lessons conducted, and the time devoted to the program, predicted reductions in self-, teacher-, and peer-reported bullying and victimization (Swift et al., 2017).

The degree to which educators implement and adhere to the protocols of AB initiatives is associated with a complex set of individual and contextual factors. Implementation, for example, is more successful when programs are supported by head teachers (Ahtola, Haataja, Kärnä, Poskiparta, & Salmivalli, 2013) and educators are confident in their ability to conduct AP programs (Boulton, 2014). Students report a greater reduction in bullying when teachers are perceived to devote more effort to bullying prevention (Veenstra, Lindenberg, Huitsing, Sainio, & Salmivalli, 2014).

Educators play a central role in the successful introduction of evidence-based programs; their preferences, therefore, should inform the design, adaptation, and implementation of school-based programs (Damschroder et al., 2009; Durlak & DuPre, 2008; Powell et al., 2015). Chorpita and colleagues concluded that, “… For treatments to be effective and sustained in practice settings, treatment developers should consider design features that increase the appeal to the therapists who are ultimately responsible for using them.” (Chorpita et al., 2015, p. 79). Educators allowed to select a practice consistent with their preferences adopted the intervention more rapidly, implemented the intervention with greater fidelity, and were more likely to sustain the intervention than the non-preference group (Johnson et al., 2014).

Several studies examined educator preferences regarding the strategies that should be included in AB programs (Bauman, Rigby, & Hoppa, 2008; Crothers & Kolbert, 2004). Educators, for example, indicated they would be most likely to employ a disciplinary strategy, enlist the intervention of other adults such as administrators and colleagues, or inform parents that the Behavior must stop (Bauman et al., 2008). In a study of the AB program design and implementation preferences of 1176 junior kindergarten to Grade 8 educators, participants preferred sustainable, universal programs linked closely to the provincial curriculum (Cunningham et al., 2009). They were sensitive to the support of students and staff and valued programs that taught AB skills to students via lectures, demonstrations, and practice. This study demonstrated the importance of individual differences in the design preferences of educators. Latent class analysis identified three classes with different design preferences: *Decision-Sensitive* educators who preferred school-based adoption decisions, *Support-Sensitive* educator*s* who preferred programs selected by local boards of education, and *Cost-Sensitive* educators who preferred to limit implementation time demands and expenses (Cunningham et al., 2009).

Identifying psychological and demographic factors associated with class membership is an important step in the conduct of latent class analyses (Berlin, Williams, & Parra, 2014; Zhou, Thayer, & Bridges, 2018). A number of studies, for example, have reported that the components of the Theory of Planned Behavior are associated with membership in latent classes preferring different approaches to the implementation of school-based mental health services (Cunningham et al., 2009, 2014). This model assumes that the intent to implement AB programs is linked to the anticipated benefits of these initiatives (Attitudes), social influences encouraging implementation (Subjective Norms), and confidence in one’s ability to conduct AB programs (Perceived Behavioral Control). Educators possessing a stronger intent to implement AB programs would be more likely to actually participate in implementation activities (Behavior). In a previous study, for example, a latent class of C*ost Sensitive* educators anticipated fewer benefits to AB programs (Attitudes), more barriers to implementation, and were less intent on participating in AB activities (Cunningham et al., 2009).

Qualitative studies also point to design and implementation factors influencing the response of educators to AB initiatives (Cunningham et al., 2016). Focus groups with 109 educators, for example, suggested that the effectiveness of AB programs was influenced by training and follow-up support, competing curriculum demands, difficulty detecting bullying incidents, ineffective responses to bullying, and administrative back-up (Cunningham et al., 2016). Educators felt frustrated by mandated AB initiatives, limited opportunities to participate in program design, inflexible protocols, and the seemingly arbitrary process via which schools replaced promising programs. Some felt cynical, limited their commitment to AB programs, selectively implemented components of AB initiatives, introduced modifications, or resisted implementation. These responses are consistent with Psychological Reactance Theory which suggests that program design and implementation processes limiting decision control might elicit responses that, although intended to retain or reassert personal agency, may undermine prevention initiatives (Brehm & Brehm, 1981; Rosenberg & Siegel, 2017). Psychological reactance has been observed in college classrooms (Ball & Goodboy, 2014), experimental prevention analogues (Legault, Gutsell, & Inzlicht, 2011), and the implementation of evidence-based practices (Gunter & Whittal, 2010). It is considered a potential challenge to the implementation of prevention programs.

### The Current Study

**Fig. 1:**
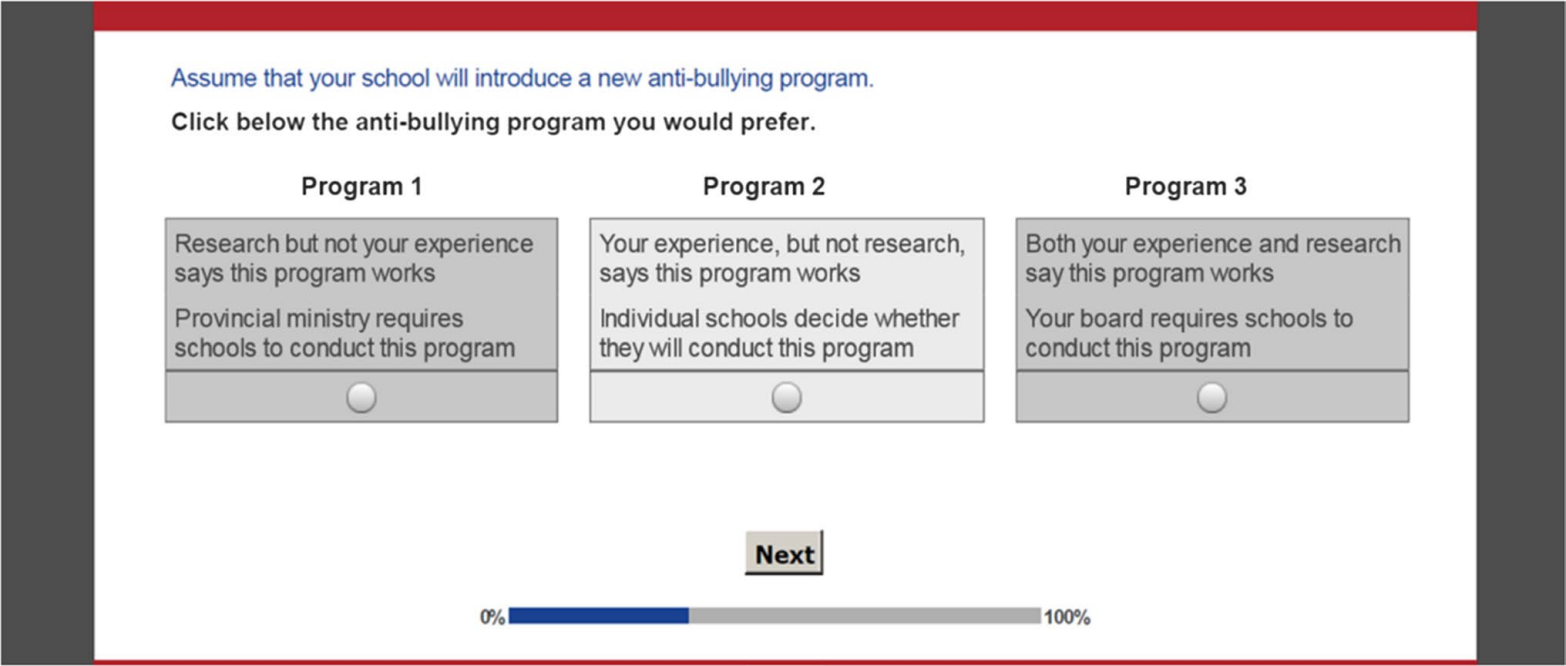
An example of the 15 choice tasks completed by each participant. Sawtooth Software’s experimental design module created 999 combinations of the survey and randomly assigned one version to each participant

This study addressed several gaps in the extant literature. First, given the role that educators play in the delivery of school-based initiatives, it is important to increase our understanding of their preference for, and response to, different approaches to the design and implementation of AB programs. The current study used a discrete choice conjoint experiment (DCE) to extend research on the AB design preferences of educators. These methods, used by marketing researchers (Orme, 2014) and health economists (de Bekker-Grob, Ryan, & Gerard, 2012), are increasingly applied to estimate the relative value of the components of school-based prevention initiatives and to “tailor” the implementation of children’s mental health services to the professionals responsible for conducting these programs (Powell et al., 2015). DCEs define educational programs as a set of features or attributes (Orme, 2014). The attributes of an AB program might include the program selection process, quality of the supporting evidence, training time demands, or number of supervisors monitoring playgrounds and hallways. Consistent with Random Utility Theory, DCEs assume that preference for an AB program is a function of the utility or value of that program’s individual attributes plus an error term (Hauber et al., 2016). Each of the attributes included in a DCE is defined by several levels. The three levels of an attribute named “selection process,” for example, might include selected by governments, selected by local boards of education, or selected by individual schools. To estimate the relative value of the attributes of a program, DCEs present choices between hypothetical programs created by experimentally combining the levels of different attributes (see Fig. 1).

DCEs can advance educational research in several ways. First, as discussed previously (Cunningham et al., 2014), choosing multi-component options approximates the complexity of real-world educational decision making (Orme, 2014; Phillips, Johnson, & Maddala, 2002); DCEs are more likely to elicit the simplifying heuristics influencing educational decisions (Hauser, 2014; Orme, 2014). Second, given the multi-component design of effective AB programs (Olweus, 1994; Ttofi & Farrington, 2011), competing curriculum demands, and cost constraints, program planners and the educators implementing AB programs must consider trade-offs. Increasing the time devoted to one component of the program, for example, may limit the time available to others. Rather than rating the individual components of complex programs, an approach allowing participants to rate all features as important, the multi-component choices presented in DCEs require participants to consider these trade-offs. Third, because informants consider each attribute of an option in context of experimentally manipulated combinations of other attributes, DCEs allow investigators to estimate the *relative influence* of each feature on AB program choices (Hauber et al., 2016; Orme, 2014). Fourth, DCEs allow educational program developers to estimate the relative importance of options that do not exist. Quantifying the relative importance of both innovative options and existing practices permits planners to simulate the response of participants to design options under consideration before costly, time-intensive implementation projects begin. Finally, responding to multi-component options in DCEs reduces the influence of the social desirability biases (Caruso, Rahnev, & Banaji, 2009; Phillips et al., 2002) that can influence the response of educators to more traditional rating scales (Larson & Bradshaw, 2017).

Second, studies using DCEs to examine the program implementation preferences of educators (Cunningham et al., 2009, 2014) have not accounted for the multi-level structure of preference data where educators are nested within schools (Vermunt, 2008). These studies risk over-estimating educator influences and neglecting potentially important school-level processes. The current study, therefore, utilized a multi-level approach to the latent class analysis of discrete choice data (Vermunt, 2008).

Third, some educators respond negatively to limitations in the opportunity to influence the selection, design, and implementation of AB programs (Cunningham et al., 2016), a response consistent with Psychological Reactance Theory (Brehm & Brehm, 1981; Rosenberg & Siegel, 2017). Although qualitative methods have provided a rich thematic account of the mechanisms via which the design and implementation of AB programs may elicit psychological reactance, their perspective is limited by small samples, self-presentation biases, difficulty quantifying the relative importance of the themes that emerge, and the absence of a mechanism for estimating the proportion of educators sharing diverging views regarding program design. We are aware of no studies quantifying this potentially important response. The current study extends previous research by asking a large sample of educators to report the extent to which they experienced psychological reactance to the AB programs in their schools. We measured a more stable tendency to resist persuasive influences (dispositional reactance) and determined the extent to which these measures were linked to membership in latent classes preferring different approaches to AB program design.

We addressed three research questions:

*RQ1. Are there latent classes preferring different approaches to AB program design?* Given previous studies (Cunningham et al., 2009), we anticipated latent classes of educators with preferences ranging from supporting the implementation of AB initiatives to a class less inclined to participate. We estimated the relative influence of 12 design attributes on the AB program choices of participants in each latent class. Based on earlier studies (Cunningham et al., 2009), we predicted that the social context in which AB programs are implemented (e.g., the response of colleagues and principals) would exert an important influence on AB program choices.

*RQ2. Is the Theory of Planned Behavior linked to latent class membership?* Psychological measures can predict and explain membership in unobserved latent classes, inform the content of advertising strategies and health communication messages, and enable implementation teams to tailor AB programs to local contexts (Zhou et al., 2018). We predicted that latent classes anticipating more benefits to AB programs (Attitudes) would be more amenable to social influences encouraging participation (Subjective Norms), express greater confidence in their ability to participate (Perceived Behavioral Control), identify fewer barriers to implementation, and reside in latent classes that were more intent on participating.

*RQ3. Is psychological reactance linked to latent class membership?* We extend qualitative research (Cunningham et al., 2016) by asking educators to indicate the extent to which they experienced different types of psychological reactance and measured variation in dispositional reactance, a more general tendency to resist persuasive efforts. We predicted that educators reporting higher dispositional reactance and greater psychological reactance to AB programs would reside in latent classes that were less intent on participating in AB activities.

## Method

### Participants

The Hamilton Integrated Research Ethics Board and the participating school boards approved this study. Educators (Table 1) were from a Canadian community of 530,000 residents. We grouped schools into five areas with differing demographics and randomly selected schools from each area. Administrators e-mailed a letter describing the study to the principals at 80 schools. Of the 70 principals we were able to contact, 48 agreed to participate. The areas of the city in which participating (*X*_P_) and nonparticipating (*X*_NP_) schools were located did not differ with respect to urban versus rural locations (*X*_p_ urban = 89.6% *X*_NP_ urban = 93.8%, *X*^2^ (1, *N* = 80) = 417, *p *= .518). Supplementary electronic Table 1 shows that median family income, adults over 25 without a high school or postsecondary diploma, percentage aged 25 to 64 with a university diploma or degree, households headed by female lone parents, or percentage of immigrants did not differ, and that regional demographics were very similar to our sample area (DeLuca, Johnston, & Buist, 2012).

**Table 1:** Demographic comparisons of the three classes of educators

	*N*	%	Latent class				
			Supervisors	Facilitators	Delegators	*χ*^2^	*df*
*Sample size*	1080	100	233	665	182		
Percentage		100.0	21.6	61.6	16.9		
*Age*						7.25	6
18–29 years	123	11.4	28.5	57.7	13.8		
30–39 years	365	33.8	21.1	60.5	18.4		
40–49 years	358	33.1	18.4	64.0	17.6		
50 years or older	234	21.7	23.5	61.5	15.0		
*Sex*						7.43^a^	2
Male	203	18.8	26.6	53.2	20.2		
Female	877	81.2	20.4	63.5	16.1		
*Education*						55.74^c^	8
High school or less	5	0.5	60.0	40.0	0.0		
College or university courses	16	1.5	56.3	43.8	0.0		
College diploma or degree	138	12.8	38.4	56.5	5.1		
Bachelor’s degree	728	67.4	19.0	62.4	18.7		
Master’s or doctoral degree	193	17.9	15.5	64.2	20.2		
*Profession*						56.57^c^	6
Administrator, Principal, or Vice Principal	38	3.5	18.4	71.1	10.5		
Teacher or learning resource teacher	869	80.5	18.1	62.3	19.7		
Educational assistants	168	15.6	39.3	56.5	4.2		
Support staff	5	0.5	60.0	40.0	0.0		
*Teaching experience (check all that apply)*							
Junior/senior kindergarten	401	37.2	20.9	67.6	11.5	14.77^b^	2
Grades 1–5	677	62.7	20.4	61.3	18.3	3.35	2
Grades 6–8	481	44.6	20.8	62.4	16.8	0.28	2
Grades 9–12	29	2.7	34.5	55.2	10.3	3.29	2
*Years of experience*						9.27	10
0–5 years	175	16.2	27.4	60.0	12.6		
6–10 years	267	24.7	20.6	61.8	17.6		
11–15 years	246	22.8	22.4	58.1	19.5		
16–20 years	161	14.9	19.3	63.4	17.4		
21–25 years	103	9.5	18.4	63.1	18.4		
More than 25 years	127	11.8	18.9	66.9	14.2		

*Supervisors* All-in Supervisors; *Facilitators* Facilitators; *Delegators* Reluctant Delegators

^a^*p* < 0.05; ^b^*p *< 0.01; ^c^*p *< 0.001

We sent links to 1313 potential participants present on the day the survey was administered at each school. All members of the school staff present (e.g., teachers, principals, educational assistants, etc.) were eligible to participate. Of those receiving the link, 12 elected not to participate, 221 discontinued the survey, and 1080 completed surveys.

### Designing the Discrete Choice Conjoint Experiment

The study’s discrete choice experiment was developed in several steps.

#### Selecting Attributes

As per guidelines, we derived attributes of AB programs via a qualitative process (Bridges et al., 2011). We conducted 19 focus groups with 103 elementary and middle school educators. Focus group methods and findings are detailed in another publication (Cunningham et al., 2016). Using Nvivo software, we coded transcripts thematically. We selected attributes that were repeatedly discussed (recurrent themes) as influencing the implementation or outcome of the AB programs in their schools. For example, the attribute “Principal Support” was discussed in 84% of the 19 focus groups. As one participant stated, “if the principal doesn’t buy into it you’re not going to get everybody on board.” (Cunningham et al., 2016). The attribute Colleague Support and Engagement was a topic of discussion in 63.2% of focus groups. For example, “There will be recidivism no matter what if you do not have the entire staff on board…” (Cunningham et al., 2016). Because the number of attributes that can be included in DCEs is limited (Bridges et al., 2011; Orme, 2014), a team with content expertise (e.g., educational researchers, school social worker, school consultant) used a consensual process to reduce potential attributes to a final set of 12. As recommended (Bridges et al., 2011; Orme, 2014), we included some attributes that proved influential in previous studies (e.g., Colleague Support and Engagement) or, like Recess Supervision, were linked to the outcome of AB programs (Ttofi & Farrington, 2011). We selected attributes that were relatively independent of one another and could be modified to improve implementation (Bridges et al., 2011; Orme, 2014). Attributes included the support and engagement of principals, colleagues, students, and parents, the extent to which programs focused on bullying versus underlying problems, supervision of playgrounds and hallways at recess, rewards for student prevention, consistent and firm consequences, the extent to which AB programming extended across grades, supporting evidence, decision control, and time for learning and implementation.

#### Specifying Attribute Levels

Each attribute was described by four levels. This design avoids a bias in favor of attributes with a greater number of levels (Orme, 2014; Wittink, Krishnamurthi, & Reibstein, 1990). For example, the levels of the attribute “Rewards Student Prevention” were: (1) rarely rewards student who prevent bullying, (2) sometimes rewards students who prevent bullying, (3) often rewards students who prevent bullying, or (4) always rewards students who prevent bullying. The survey was piloted to ensure that attributes were easily understood and combined logically in choice tasks (Orme, 2014). Attributes and their levels are presented in Table 2.

**Table 2:** Zero-centered utility coefficients and *Z* value comparisons for the three classes of educators

	Latent class						*Wald*
Attribute	Supervisors		Facilitators		Delegators		
Content of attribute levels	*U*	*Z*	*U*	*Z*	*U*	*Z*	
*Principal Support*							172.64^c^
Principal does not champion this program and does not back teachers up	− 0.61	− 5.68	− 1.95	− 14.92	− 1.17	− 5.85	
Principal champions this program but does not back teachers up	− 0.22	− 2.37	− 1.06	− 10.14	− 0.89	− 5.00	
Principal does not champion this program but does back teachers	− 0.15	− 1.53	0.75	9.42	0.31	2.22	
Principal champions this program and backs teachers up	**0.98**	11.13	**2.26**	25.56	**1.75**	12.01	
*Consistency across grades*							50.43^c^
Runs in kindergarten but discontinued in Grade 1	− 1.04	− 7.61	− 2.14	− 16.90	− 1.33	− 6.67	
Runs from kindergarten to Grade 5 and then discontinued	− 0.61	− 5.14	− 0.44	− 5.27	− 0.14	− 0.99	
Runs from kindergarten to Grade 8 and then discontinued	0.54	5.60	0.98	13.87	0.44	3.36	
Runs from kindergarten to Grade 12	**1.11**	11.14	**1.59**	20.66	**1.03**	7.47	
*Student engagement in AB programs*							164.24^c^
Students resist participating in this program	− 0.75	− 6.90	− 1.62	− 13.50	− 1.69	− 7.08	
Students just go through the motions with this program	− 0.45	− 4.22	− 1.68	− 13.97	− 0.67	− 3.54	
Students actively participate in this program	**0.74**	8.33	1.53	18.91	1.14	7.94	
Students take ownership of this program	0.47	4.94	**1.77**	21.10	**1.21**	7.78	
*Colleague Support and Engagement*							138.48^c^
Your colleagues don’t like or participate in this program	− 0.48	− 4.60	− 1.36	− 14.84	− 1.07	− 6.03	
Your colleagues like but don’t participate in this program	− 0.12	− 1.21	− 0.47	− 5.93	0.21	1.59	
Your colleagues participate in but don’t like this program	− 0.13	− 1.35	− 0.17	− 2.14	− 0.55	− 3.44	
Your colleagues like and participate in this program	**0.73**	7.87	**1.99**	26.43	**1.41**	9.79	
*Consistent and firm consequences*							184.90^c^
Consequences are not consistent for all students nor firm enough	− 0.27	− 2.78	− 1.78	− 15.61	− 0.97	− 5.61	
Consequences are consistent for all students but not firm enough	− 0.20	− 2.13	− 0.01	− 0.18	− 0.73	− 4.71	
Consequences are firm enough but not consistent for all students	0.04	0.46	− 0.10	− 1.19	0.39	2.94	
Consequences are firm enough and consistent for all students	**0.42**	4.42	**1.89**	23.14	**1.30**	8.84	
*Focus on underlying problems versus bullying*							73.76^c^
Focuses 100% on problems underlying bullying and 0% on bullying	− 0.06	− 0.62	− 0.86	− 9.44	− 0.44	− 2.68	
Focuses 67% on problems underlying bullying and 33% on bullying	**0.68**	7.45	**1.36**	18.66	**0.57**	3.97	
Focuses 33% on problems underlying bullying and 67% on bullying	0.44	4.76	0.87	12.15	0.43	3.03	
Focuses 0% on problems underlying bullying and 100% on bullying	− 1.06	− 8.16	− 1.37	− 14.27	− 0.56	− 3.31	
*Supporting evidence*							126.34^c^
Neither your experience nor research says this program works	− 0.08	− 0.88	− 1.33	− 13.37	− 0.78	− 4.97	
Research but not your experience says this program works	− 0.15	− 1.66	− 0.21	− 2.76	− 0.24	− 1.75	
Your experience, but not research, says this program works	0.02	0.27	0.12	1.73	0.25	1.93	
Both your experience and research say this program works	**0.21**	2.30	**1.41**	18.81	**0.77**	6.14	
*Parental support and engagement*							72.02^c^
Parents don’t participate in or like this program	− 0.33	− 3.24	− 1.19	− 12.99	− 0.88	− 5.61	
Parents participate in this program but don’t like it	− 0.27	− 2.78	− 0.36	− 4.83	− 0.35	− 2.36	
Most parents like this program but don’t participate	0.15	1.61	0.23	3.32	0.09	0.76	
Most parents participate in and like this program	**0.46**	5.08	**1.33**	18.94	**1.13**	8.21	
*Time for learning and implementation*							234.27^c^
You don’t have enough time to fully learn or fully implement this program	**0.23**	2.58	− 1.43	− 13.25	− 0.89	− 5.30	
You have enough time to fully implement but not to fully learn this program	− 0.30	− 3.06	− 0.20	− 2.60	− 0.15	− 1.03	
You have enough time to fully learn this program but not to fully implement	0.03	0.33	0.05	0.75	− 0.14	− 0.95	
You have enough time to fully learn and fully implement this program	0.04	0.42	**1.57**	20.23	**1.17**	7.66	
*Rewards for student prevention*							64.29^c^
Rarely rewards students who prevent bullying	− 0.46	− 4.60	− 1.30	− 14.20	− 0.43	− 2.78	
Sometimes rewards students who prevent bullying	− 0.06	− 0.61	− 0.12	− 1.70	0.13	1.05	
Often rewards students who prevent bullying	0.01	0.14	**0.72**	10.50	**0.24**	1.91	
Always rewards students who prevent bullying	**0.51**	5.75	0.70	9.95	0.05	0.37	
*Recess Supervision*							361.14^c^
Non-teaching staff supervise playgrounds and hallways at every recess	− 0.68	− 5.05	− 0.75	− 6.94	**2.23**	11.40	
25% of educators must supervise playgrounds and hallways at every recess	0.04	0.42	**0.93**	12.56	0.93	4.67	
50% of educators must supervise playgrounds and hallways at every recess	0.24	2.62	0.37	5.04	− 0.92	− 4.28	
All educators must supervise playgrounds and hallways at every recess	**0.39**	3.92	− 0.55	− 5.86	− 2.25	− 5.41	
*Decision control*							25.17^c^
Provincial ministry requires schools to conduct this program	− 0.11	− 1.20	− 0.24	− 3.34	− 0.22	− 1.44	
Your board requires schools to conduct this program	− 0.01	− 0.15	0.21	3.15	− 0.54	− 3.31	
Individual schools decide whether they will conduct this program	**0.18**	1.97	**0.34**	5.09	**0.48**	3.31	
Individual educators decide whether they will conduct this program	− 0.05	− 0.55	− 0.32	− 4.36	0.28	1.90	

For each attribute, the highest utility for each segment is bolded. Utility coefficients with *Z* values > 1.95 differ significantly from zero

*Wald* measures the statistical significance of the differences in the utility coefficients of the three classes; *Supervisors* All-in Supervisors; *Facilitators* Facilitators; *Delegators* Reluctant Delegators

^c^*p *< 0.001

#### Designing Choice Sets

Sawtooth Software’s experimental design algorithm created 999 sets of choice tasks with different combinations of the study’s attribute levels (Johnson et al., 2013). One set was randomly assigned to each participant. Each choice set presented a warm-up choice task introducing this method, 15 experimental choice tasks (see Fig. 1), and two hold-out choice tasks to examine internal validity. Each choice task presented three hypothetical AB programs. According to a partial profile experimental design, each AB program was described by the levels of two of the study’s 12 attributes (Chrzan, 2010; Orme, 2014). Rather than presenting choices between options described by 12 attributes, partial profile designs present choices between options described by a subset of the study’s attributes. By simplifying choice tasks, partial profile designs reduce the impact of dominant attributes, encourage participants to weigh the incremental contribution of less important features of the program, and improve predictive validity (Chrzan, 2010). Participants were instructed to, “Assume that your school will introduce a new anti-bullying program. Click below the anti-bullying program you would prefer.”

### Measuring Factors Linked to Latent Class Membership

#### Theory of Planned Behavior

To address RQ2, we composed a set of questions based on the Theory of Planned Behavior (Ajzen, 1991), a model that has been linked to the implementation of AB programs in previous studies (Cunningham et al., 2009). Participants completed the five-point Likert questions (1 = Strongly Disagree to 5 = Strongly Agree) described below. To measure *Attitudes*, five questions examined the anticipated benefits of AB programs (e.g., Reduce the number of students who are victims of bullying), *α* = 0.93. To measure *Subjective Norms*, six questions measured the influence of different individuals and organizations on the decision to participate in AB programs (e.g., My teaching colleagues; The principal at my school), *α* = 0.89. To measure *Perceived Behavioral Control,* five questions examined confidence in one’s ability to implement and conduct AB programs (e.g., I have the confidence to prevent or respond to bullying), *α* = 0.89. *Barriers*, a component of Perceived Behavioral Control, was measured by five questions describing factors that may compromise implementation (e.g., Too many other programs to conduct), *α* = 0.84. Although barriers are typically considered a component of Perceived Behavioral Control, studies finding that this measure contributed independently to the prediction of intentions (and Behavior) recommended measuring barriers separately (Bozionelos & Bennett, 1999). Six questions measured the *Intent* to participate in different activities linked to the implementation of AB programs (e.g., I would be willing to participate in a 1-day workshop teaching skills to prevent bullying), *α* = 0.79. For all scales, responses to individual questions were summed to yield a total score.

#### Psychological Reactance

To address RQ3, eight questions (1 = never, 2 = once a year, 3 = once a month, 4 = once a week, and 5 = once a day) derived from focus groups (Cunningham et al., 2016) measured cognitive, affective, and behavioral responses consistent with Psychological Reactance Theory (e.g., Felt cynical about AB programs; Told your colleagues AB programs are not working). Responses to individual questions were summed to yield a total score, *α* = 0.90.

#### Dispositional Reactance

The 14-question (1 = Strongly Disagree, 5 = Strongly Agree) Hong Dispositional Reactance Scale (Shen & Dillard, 2005) measured a tendency to resist persuasive efforts (e.g., “I resist the attempts of others to influence me”). Responses to individual questions were summed to yield a total score, *α* = 0.89.

#### Demographics

Respondents recorded their sex, years of educational experience, experience teaching different grades, etc. (Table 1).

### Procedure

After endorsing an electronic consent, participants completed anonymous online surveys on computers at their schools. The software did not record IP addresses. Participants read the provincial Ministry of Education’s definition of bullying, answered Theory of Planned Behavior questions, and responded to DCE choice tasks, demographic questions, and measures of psychological reactance. Those completing the survey were given the option of entering a draw for one of the twelve $50.00 gift certificates to a national bookstore. Median time to complete the survey was 16.6 min.

### Data Analysis

#### Fitting a Latent Class Model

To address RQ1, we used multi-level latent class analysis (Latent Gold Choice 5.1) to estimate a three-level model (Vermunt, 2008). To enable multi-level latent class analyses, we generated an anonymous code for the surveys in each school. The actual identify of the school was not linked to survey data. The 15 choices (Level 1) were nested within educators (Level 2), who were nested within schools (Level 3) (Vermunt, 2008). At Level 2, we estimated discrete random effects models specifying from 1 to 8 latent classes of educators (Hauber et al., 2016). At Level 2, latent classes comprise clusters of *educators* preferring different approaches to the design or implementation of AB programs. Next, we estimated from 1 to 3 latent classes of schools (Level 3) as discrete random effects (Vermunt, 2008). Latent classes at Level 3 comprise clusters of schools in which the proportion of educators in Level 2 latent classes preferring different approaches to the design of AB programs varies. For example, one Level 3 latent class of schools might have a greater proportion of educators in Level 2 latent classes preferring that individual schools select AB programs. Another Level 3 latent class might have a greater proportion of educators in latent classes preferring that the ministry of education selects AB programs. To avoid an unrepresentative local solution, we computed each model 250 times from semi-random starting points and retained the best fitting model (Berlin et al., 2014; Hauber et al., 2016; Nylund, Asparouhov, & Muthén, 2007; Vermunt & Magidson, 2005). Decisions regarding the number of latent classes to retain at Levels 2 and Level 3 were based on fit indices (e.g., Bayesian Information Criterion (BIC)), latent class size, and conceptual utility (Berlin et al., 2014; Lanza & Rhoades, 2013; Zhou et al., 2018). Educators at Level 2 and schools at Level 3 were assigned to a latent class with the highest posterior probability of group membership (Hauber et al., 2016; Vermunt, 2008; Zhou et al., 2018).

#### Estimating Utility Coefficients

Analysis integrated latent class and conditional logit to fit zero-centered utility coefficients to effects-coded data for each latent class (Hauber et al., 2016; Vermunt, 2008; Zhou et al., 2018). Higher utility coefficients reflect a stronger preference in comparison with other levels of that attribute.

#### Calculating Relative Importance Scores

We derived importance scores reflecting the proportion of variation in Level 2 utility coefficients accounted for by variation in the levels of each attribute (Orme, 2014; Vermunt, 2008). For each latent class, the range of each attribute’s utility coefficients (high minus low) was summed to yield a total range. Each attribute’s range was divided by the total range and multiplied by 100 to yield a percentage. Higher importance scores mean that variation in the levels of an attribute exerted a greater influence on program choices.

#### Identifying Factors associated with Latent Class Membership

To address RQ2 and RQ3, we computed one-way multivariate analysis of variance (MANOVAs) determining whether the components of the Theory of Planned Behavior and measures of psychological and dispositional reactance differed as a function of latent class membership. When overall tests in MANOVAs were significant, we computed univariate analysis of variance (ANOVAs) followed by post hoc Dunnett’s C comparisons. The criterion for statistical significance was set apriori at alpha = 0.05. These analyses were performed using SPSS version 25.

## Results

*RQ1. Are there latent classes of educators preferring different approaches to AB program design?* A three-class solution (Supplementary Table 2) yielded the lowest BIC, classes with a relatively large number of participants, and a conceptually useful model (Lanza & Rhoades, 2013). Although the addition of two school-level classes to the model increased BIC, it reduced Akaike Information Criterion (AIC), a less conservative fit index (Dziak, Coffman, Lanza, Li, & Jermiin, 2019). We labeled the classes as *All*-*in Supervisors* (21.5%), *Facilitators* (61.6%), and *Reluctant Delegators* (16.9%). Multi-level findings (Supplementary Electronic Table 3) show the probability that the educators in each of Level 3’s two classes of schools were members of Level 2’s three classes of educators. At the level of schools, a two-class solution assigned 61% of schools to Class 1 and 39% of schools to Class 2. In comparison with school Class 2, a greater proportion of school Class 1’s educators (26% vs. 3%) were *Reluctant Delegators*. A greater proportion of Class 2’s educators were *Facilitators* (69% vs. 52%). Zero-centered utility coefficients are presented in Table 2 in order of their relative importance to *All*-*in Supervisors.* Supplementary Electronic Table 4 shows that educators in the Level 3 class of schools with a greater proportion of Reluctant Delegators (school Class 1) reported higher dispositional reactance, more psychological reactance, were less likely to be influenced by social norms, and less confident in their ability to implement AB programs.

*All-in Supervisors (21.5%).* This class preferred that 100% of educators supervised playgrounds and hallways at every recess (Table 2). They preferred that students actively participate in the program and that those preventing bullying were always rewarded. Consistency across grades and a focus on both bullying and the underlying contributors to this problem were particularly important to this class (Table 3). The quality of the evidence supporting program effectiveness, in contrast, exerted little influence on their choices.

**Table 3:** Relative importance of AB program design attributes to three classes of educators

	Latent class					
	Supervisors		Facilitators		Delegators	
Attributes	*R*	*I*	*R*	*I*	*R*	*I*
Consistency across grades	**1**	**16.7**	2	11.1	5	9.1
Focus on underlying problems versus bullying	2	**13.5**	8	8.1	10	4.4
Principal support	3	12.4	**1**	**12.5**	2	11.3
Student engagement in AB programs	4	**11.6**	4	10.2	3	11.2
Colleague support and engagement	5	9.4	5	**9.9**	4	9.6
Recess Supervision	6	8.3	11	5.0	**1**	**17.3**
Rewards for student prevention	7	**7.5**	10	6.0	12	2.6
Parental support and engagement	8	6.1	9	7.5	8	**7.8**
Consistent and firm consequences	9	5.3	3	**10.9**	6	8.8
Time for learning and implementation	10	4.2	6	**8.9**	7	8.0
Supporting evidence	11	2.8	7	**8.1**	9	6.0
Decision control	12	2.3	12	2.0	11	**3.9**

Attributes are ranked in order of their importance to the All-in Supervisors class. *R* Rank of each attribute’s importance within each class; *I* Relative importance of each attribute expressed as a percentage of the total variability (high to low) across utility coefficients. Within each class, importance scores add to 100.0 with the highest score for each attribute bolded. Variation in the levels of attributes with higher importance scores exerts a greater influence on program design choices. *Supervisors* All-in Supervisors; *Facilitators* Facilitators; *Delegators* Reluctant Delegators

*Facilitators (61.6%). Facilitators* preferred that 25% of educators supervise playgrounds and hallways with students taking ownership of AB programs (Table 2). They preferred that students preventing bullying were often rewarded. Programs extending from kindergarten to Grade 12 exerted more influence on choices than any other design attribute (Table 3). Time for learning and implementation exerted a moderately important influence on program choices (Table 3).

*Reluctant Delegators (16.9%). Reluctant Delegators* preferred that non-teaching staff supervised playgrounds and hallways (Table 2). Variation in responsibility for playground and hallway supervision exerted a greater influence on this class’s choices than any other design attribute (Table 3). Although they preferred that students took ownership of the program, rewarding those who prevented bullying was of lower importance than any other attribute.

*Shared preferences.* Educators agreed on a significant proportion of the study’s design features. Variation in the support and engagement of principals, colleagues, and students exerted an important influence on program choices of all classes of educators (Table 3). All classes preferred programs championed by principals who backed teachers up (Table 2). Educators preferred principals who backed them up more than those who simply championed programs. Educators were more likely to choose AB programs which colleagues, students, and to a lesser extent parents liked and participated in. When considering the levels of parental involvement, educators valued programs that parents liked more than those that parents participated in. All classes preferred AB programs running from kindergarten through Grade 12. Educators chose programs supported by both personal experience and research. Given a choice between programs backed by research versus those supported by personal experience, however, most would base decisions on their experience. All classes preferred AB programs that focused 67% on problems underlying bullying and 33% on bullying incidents. They advocated consequences that were firm enough and consistent for all students. Although they preferred that individual schools rather than school boards or government ministries of education make decisions about the adoption of AB programs, importance scores suggest that decision control exerted a relatively limited influence on program choices (Table 3).

*RQ2. Is the Theory of Planned Behavior linked to latent class membership?* A MANOVA across the Theory of Planned Behavior’s constructs showed a significant class effect, *F*(10, 2148) = 6.86, *p *< .001*. Reluctant Delegators* reported more barriers to the implementation of AB programs than did *All*-*in Supervisors* or *Facilitators* (Table 4). *Facilitators* reported their decision to participate in AB programs was more likely to be influenced by individuals and organizations (Subjective Norms) than did *All*-*in Supervisors* or *Reluctant Delegators*. *Reluctant Delegators* intended to participate in fewer AB activities than *All*-*in Supervisors* who intended to participate in fewer AB activities than *Facilitators*. Educators in the Level 3 class of schools with a greater proportion of Reluctant Delegators (school Class 1) reported significantly fewer benefits to AB programs (Attitudes), were less likely to be influenced by social norms, and less confident in their ability to implement AB programs (Perceived Behavioral Control). Although these differences were statistically significant, effect sizes were small (Supplementary Electronic Table 4).

**Table 4:** Theory of Planned Behavior and psychological reactance scales comparisons for the three classes

Variable	Latent class						*F*	*p*	*C*	*η*^2^
	Supervisors		Facilitators		Delegators					
	M	SD	M	SD	M	SD				
*Theory of Planned Behavior*										
Attitudes	19.28	4.48	19.69	3.90	19.12	3.63	1.95	0.14		0.004
Subjective norms	21.83	5.28	22.79	4.14	21.28	4.60	9.96	< 0.001	*F* > *S*, *D*	0.018
Perceived behavioral control	16.67	4.08	16.49	3.92	16.09	4.21	1.14	0.32		0.002
Barriers	18.10	4.25	18.28	4.11	19.42	3.80	6.51	< 0.01	*D* > *F*, *S*	0.012
Behavioral intention	19.26	4.53	20.14	4.29	17.53	4.89	25.05	< 0.001	*F* > *S* > *D*	0.044
*Psychological reactance*										
Psychological reactance	13.79	6.56	13.06	5.68	14.69	6.33	5.63	< 0.01	*D* > *F*	0.010
Dispositional reactance	36.39	9.42	34.46	8.26	37.09	7.86	9.33	< 0.001	*D*, *S* > *F*	0.017

*Supervisors* All-in Supervisors; *Facilitators* Facilitators; *Delegators* Reluctant Delegators; *C* post hoc Dunnett’s C comparisons; *η*^*2*^ = Partial *η*^2^ .01 = small, .06 = medium, .14 = large effect size

*RQ3. Is psychological reactance linked to latent class membership?* A MANOVA across dispositional reactance and psychological reactance measures showed a significant class effect *F*(4, 2154) = 6.23, *p *< .001. *Reluctant Delegators* reported higher dispositional reactance than did *Facilitators* or *All*-*in Supervisors* (Table 4). *Reluctant Delegators* also reported engaging in or experiencing more psychological reactance to AB programs than did *Facilitators*. Educators in the Level 3 class of schools with a greater proportion of Reluctant Delegators (Class 1) reported higher dispositional reactance and psychological reactance scores (Supplementary Electronic Table 4). Although these differences were statistically significant, effect sizes were small.

## Discussion

This study makes three contributions to the study of the AB program design preferences of educators. First, we illustrate the use of preference modeling strategies from marketing research (Orme, 2014) and health economics (de Bekker-Grob et al., 2012) to engage educators in the AB program design process. These methods, which are relatively new to the study of school-based programming, allowed us to estimate the relative importance of the individual components of hypothetical AB programs, identify latent classes preferring a different approach to program design, and identify correlates of segment membership. Second, we extend previous studies by applying a multi-level latent class approach to the analysis of choice data (Vermunt, 2008; Zhang, Zhang, Zhang, & Jiao, 2014). Multi-level analysis points to a potentially important clustering of *Reluctant Delegators* in one class of schools. Third, this is, to our knowledge, the first study to establish empirical links between measures of dispositional and psychological reactance and membership in classes preferring different approaches to the design of AB programs. Below we consider the applied implications of our findings, revisit focus group discussions conducted prior to this study for suggestions as to why those attributes were important to educators (Cunningham et al., 2016), and examine empirical evidence regarding the impact of this set of attributes on the implementation process.

### Summary and Implications

#### Ensure Supportive Principals

Principals exerted an important influence on AB program choices. Focus groups suggested that principal buy-in encouraged the level of staff participation needed to conduct AB programs (Cunningham et al., 2016). In a longitudinal study of the KiVa program, for example, teachers who evidenced high or moderate adherence, coupled with principals perceived to support the program, were more likely to remain adherent throughout the year (Haataja, Ahtola, Poskiparta, & Salmivalli, 2015). A study of Finnish educators found that perceived head teacher support for the implementation of the KiVa AB program was associated with greater adherence (Ahtola et al., 2013). Adherence and program dosage, in turn, have been associated with classroom reductions in victimization (Haataja et al., 2014; Swift et al., 2017).

Educators preferred principals who provided back-up, rather than simply championing AB programs. Focus groups suggested that principal back-up was particularly important when dealing with confrontational students or parents (Cunningham et al., 2016). In a national sample of 2998 educators, 80% had been the targets of harassment, bullying, or violence, many by students and parents (Reddy et al., 2013). The bullying and interpersonal aggression directed at educators predicts burnout and emotional exhaustion, factors that may adversely affect the implementation of AB programs, and, ultimately, decisions to leave the profession (Reddy et al., 2013).

#### Ensure the Support of Colleagues

Consistent with RQ1’s predictions, the support and engagement of colleagues exerted an important influence on AB program choices. In focus groups, educators suggested that having the entire staff “on board” was critical to the successful implementation of AB programs. This is consistent with previous studies (Cunningham et al., 2009, 2014) and a broader body of implementation science (Damschroder et al., 2009; Durlak & DuPre, 2008). All segments preferred that colleagues both liked and participated in the program. Results, however, suggest that schools may have particular difficulty securing the support of *Reluctant Delegators.* This class reported more barriers to implementation, more dispositional reactance, greater reactance to AB programs, and a lower intent to participate in AB activities. Although *Reluctant Delegators* constituted a relatively small class (16.9%), the sensitivity of educators to the views of their colleagues (Cunningham et al., 2009) suggests that they might exert a significant influence on implementation decisions. Multi-level analysis finding that the concentration of *Reluctant Delegators* in one of the two school-level latent classes (26% vs. 3%) suggests their influence might vary across schools. Educators in the Level 3 class of schools with a greater proportion of Reluctant Delegators (Class 1) reported higher dispositional reactance, more psychological reactance, were less likely to be influenced by social norms, and less confident in their ability to implement AB programs.

The preferences of *Reluctant Delegators* are similar to those of a previous study’s *Cost-Sensitive* class who identified more barriers to the implementation of AB programs and were less intent on participating (Cunningham et al., 2009). The stability of this three-segment latent class finding is striking. Although we recruited a new sample of educators, introduced new attributes, and incorporated levels focusing on different design issues, both studies revealed three latent classes with a small group of educators (17% here, 16% previously) who seemed hesitant to support the implementation of AB programs(Cunningham et al., 2009).

How should schools engage *Reluctant Delegators*? This class valued many of the design attributes preferred by *Facilitators* and *All-in Supervisors*: principals who championed programs and backed teachers up and colleagues who liked and participated in AB programs. The Theory of Planned Behavior suggests that reducing implementation barriers and enhancing the influence of significant colleagues and administrators would increase the intent to participate. Psychological Reactance Theory, moreover, suggests that enhancing participation during the design and implementation process (Legault et al., 2011), coupled with programming encouraging an empathic response to this problem (Shen, 2010), would encourage implementation.

#### Engage Students and Parents

Student engagement exerted an important influence on AB program preferences. Focus groups suggested that, in the absence of the cooperation of students and parents, it was difficult to deal with bullying incidents (Cunningham et al., 2016). In a previous study, educators were more likely to choose AB programs supported by a clear majority of their students (Cunningham et al., 2009). Enlisting student bystanders is a central component of programs such as KiVa which have proved effective in reducing bullying (Salmivalli, Kärnä, & Poskiparta, 2010). Although student engagement exerted an important influence on AB design preferences, mobilizing parental support and engagement was of relatively low importance to educators. Systematic reviews, nonetheless, suggest that the inclusion of parents via parent training or meetings is associated with a greater reduction in bullying and victimization (Ttofi & Farrington, 2011).

#### Ensure Continuity

Consistent with previous studies (Cunningham et al., 2009, 2016), participants preferred AB initiatives providing stable programming from kindergarten to Grade 12. Educators participating in focus groups expressed concern regarding a tendency for schools to discontinue programs in favor of new initiatives without giving potentially effective programs time to work (Cunningham et al., 2016). The importance of program stability is supported by meta-analyses finding the duration of AB programs to be associated with improved outcome (Ttofi & Farrington, 2011). This attribute’s high importance suggests that educators may be more likely to invest the time and effort needed to implement AB programs successfully when they are confident these initiatives will be sustained.

#### Accommodate Differing Views Regarding the Supervision of Playgrounds and Hallways

In focus groups, educators reported difficulty detecting bullying on the playgrounds and in the hallways where these incidents occur (Cunningham et al., 2016). Vaillancourt and colleagues (2010) advocated an increase in the number of adults supervising students in high-risk areas. Systematic reviews confirm that increased supervision is associated with lower bullying and victimization (Ttofi & Farrington, 2011). The three classes, however, brought a different perspective to Recess Supervision. *All*-*in-Supervisors* thought all educators should be engaged in supervision, *Facilitators* preferred that only 25% of educators provided supervision, and *Reluctant Delegators* preferred this responsibility was shifted to non-teaching staff. Importance scores suggest the allocation of supervisory responsibilities exerted a stronger influence on the choices of *Reluctant Delegators* than any other attribute. The sensitivity of *Reluctant Delegators* to the allocation of supervisory responsibilities emphasizes the importance of an approach to the implementation of playground monitoring strategies that engages this class.

#### Balanced Focus on Bullying and Underlying Problems

Educators preferred programs focusing on the underlying problems contributing to bullying. In focus groups, for example, educators discussed the influence the peer group processes that limit the extent to which students act empathically or discourage students from participating in AB program (Cunningham et al., 2016). Educators also valued AB programs with consequences that were firm and consistent for all students. The importance of effective consequences was a recurrent theme in focus group discussions (Cunningham et al., 2016) and is consistent with systematic reviews (Ttofi & Farrington, 2011). A study of Grade 9 students reported that bullying was less frequent in schools that consistently enforced rules in the context of caring and respectful interactions with teachers (Gregory et al., 2010).

#### Provide Evidence of Efficacy and Effectiveness

Program choices were influenced by both research and personal experience supporting the effectiveness of AB initiatives. Experience exerted more influence on choices than scientific studies. Because teachers observe few bullying episodes (Craig, Pepler, & Atlas, 2000), they may have difficulty evaluating the impact of the AB programs in their schools. Focus group participants, for example, suggested that they were not provided with convincing evidence regarding program effectiveness or that their observations provided little evidence that bullying was declining (Cunningham et al., 2016). The perception that programs are effective is important; educators who judged the KiVa program to be more effective were more likely to be members of a latent class showing high implementation (Haataja et al., 2015). By supplementing research evidence with the student-reported school climate surveys available in many jurisdictions, Boards of Education could provide local outcome data that approximate the experiential evidence that educators valued.

#### Engage Educators in Decision Making

All classes preferred school-based decisions regarding the adoption of AB programs. This is consistent with both qualitative (Cunningham et al., 2016) and quantitative studies (Cunningham et al., 2009, 2014). Focus groups thought that participatory decisions promoted the consensus needed to support implementation and decreased the pushback which may result from top-down adoption processes (Cunningham et al., 2016). In a sample of 544 US schools, a local program selection process was associated with greater implementation intensity (Payne, Gottfredson, & Gottfredson, 2006). The choices of *Reluctant Delegators* were more sensitive to variations in the program selection process than were those of *Facilitators* and *All*-*in Supervisors*. Given higher dispositional reactance, more psychological reactance, a perception of more barriers to the implementation of AB programs, and a lower intent to participate, it may be particularly difficult to engage *Reluctant Delegators* in the planning process. Random Utility Theory suggests that AB programs ensuring the inclusion of high value attributes would compensate for the disutility attributable to a mandated program selection process.

## Limitations

The results of this study need to be considered in the context of several limitations. First, this research was conducted in a unionized public educational system located in an economically and culturally diverse Canadian community. The generalizability of these findings is unclear. Second, although only 48 of the 70 schools identified as potential recruiting sites participated, the demographics of the general areas in which participating and nonparticipating schools were located did not differ significantly. Third, our approach to anonymous coding of schools did not allow us to link school demographics to Level 3 class membership. Describing the characteristics of schools in latent classes at Level 3 would be an important direction for future studies. Fourth, we studied the influence of 12 4-level AB program design and implementation attributes that emerged as recurrent themes from focus groups with educators. Our models are limited by attributes that were not included. Last, although we report good internal consistency for the Theory of Planned Behavior and psychological reactance scales, a more detailed presentation of the psychometric properties of these scales goes beyond the current manuscript.

## Conclusion

Educators agree on the importance of contextual support, student engagement, firm and consistent consequences, and stable programming. These design preferences are supported by systematic reviews and implementation research. Latent class analysis points to classes of educators with diverging views regarding strategically important dimensions of program design, differences in Attitudes that may influence the intent to participate in or react to program implementation, and the potential clustering of classes of educators within schools.

## Electronic supplementary material

Below is the link to the electronic supplementary material.

Supplementary material 1 (DOCX 25 kb)

Supplementary material 2 (DOCX 28 kb)

Supplementary material 3 (DOCX 25 kb)

Supplementary material 4 (DOCX 27 kb)
