# Supplementary material for: What Influences Educators’ Design Preferences for Bullying Prevention Programs? Multi-level Latent Class Analysis of a Discrete Choice Experiment
Source: School Ment Health. 2019 Jun 22;12(1):22–37. doi: 10.1007/s12310-019-09334-0 (PMC7021664; doi:10.1007/s12310-019-09334-0)
Supplement: Supplementary file 1 — Supplementary material 1 (DOCX 25 kb) [file 12310_2019_9334_MOESM1_ESM.docx]

Supplementary Electronic Table 1

*Demographic Characteristics of Area in Which Participating and Nonparticipating Schools were Located and for Entire region*

|  | Region | Xp | | Xnp | |  |  | |  |
| --- | --- | --- | --- | --- | --- | --- | --- | --- | --- |
|  | M | M | SD | M | SD | T | *p* | |  |
| Median family income | 71,989 | 69,510 | 19,411 | 71,764 | 21,227 | -0.490 | | 0.625 | |
| **Percentage of** |  |  |  |  |  |  |  | |  |
| Adults 25-64 with no high school or postsecondary diploma | 13.0 | 15.6 | 7.4 | 14.7 | 9.0 | 0.515 | 0.608 | |  |
| Adults 25-64 with a university diploma/degree | 22.0 | 18.3 | 10.3 | 23.0 | 17.0 | 1.521 | 0.132 | |  |
| Female lone-parent families | 14.0 | 14.0 | 6.3 | 15.2 | 7.5 | -0.747 | 0.457 | |  |
| Immigrants | 24.0 | 25.3 | 9.0 | 24.5 | 8.5 | 0.389 | 0.699 | |  |

*Note.* Derived from (DeLuca et al., 2012). X_P_ = Participating schools, X_NP_ = Nonparticipating schools.
