# Supplementary material for: What Influences Educators’ Design Preferences for Bullying Prevention Programs? Multi-level Latent Class Analysis of a Discrete Choice Experiment
Source: School Ment Health. 2019 Jun 22;12(1):22–37. doi: 10.1007/s12310-019-09334-0 (PMC7021664; doi:10.1007/s12310-019-09334-0)
Supplement: Supplementary file 2 — Supplementary material 2 (DOCX 28 kb) [file 12310_2019_9334_MOESM2_ESM.docx]

Supplementary Electronic Table 2

*Fit Statistics for Multi-Level Latent Class Analysis*

| Model | Npar | Log-likelihood (LL) | Log-prior | Log-posterior | AIC (based on LL) | AIC3 (based on LL) | BIC (based on LL) | CAIC (based on LL) | Entropy R^2^ |
| --- | --- | --- | --- | --- | --- | --- | --- | --- | --- |
| 1 class of teachers | 36 | -12491.7 | -1.6 | -12493.2 | 25055.38 | 25091.38 | 25234.83 | 25270.83 | 1 |
| 2 classes of teachers | 73 | -12142.0 | -2.4 | -12144.4 | 24430.00 | 24503.00 | 24793.88 | **24866.88** | 0.65 |
| 3 classes of teachers | 110 | -12002.8 | -2.9 | -12005.7 | 24225.66 | 24335.66 | **24773.98** | 24883.98 | 0.63 |
| 4 classes of teachers | 147 | -11896.4 | -3.2 | -11899.5 | 24086.71 | 24233.71 | 24819.46 | 24966.46 | 0.63 |
| 5 classes of teachers | 184 | -11813.4 | -3.4 | -11816.8 | 23994.76 | 24178.76 | 24911.95 | 25095.95 | 0.63 |
| 6 classes of teachers | 221 | -11737.6 | -4.2 | -11741.8 | **23917.25** | **24138.25** | 25018.88 | 25239.88 | 0.65 |
| 3 classes of teachers  2 Level 3 classes of schools | 149 | -11949.4 | -3.8 | -11953.2 | 24196.76 | 24345.76 | 24939.48 | 25088.48 | **0.67** |
| 3 classes of teachers  3 Level 3 classes of schools | 188 | -11919.7 | -4.1 | -11923.7 | 24215.36 | 24403.36 | 25152.49 | 25340.49 | 0.63 |

*Note.* Entropy R^2^ reflects the degree to which classes are separated. Scores range from 0 to 1 with higher scores reflecting greater separation; Npar = number of parameters estimated; AIC = Akaike Information Criterion; BIC = Bayesian Information Criterion; CAIC = Consistent Akaike Information Criterion. For the information criteria (AIC, AIC3, BIC. CAIC), the model with the best fit is bolded. Note that decisions regarding the retention of latent classes are typically based on a set of factors that could include statistical fit, class size, explanatory value, and administrative utility (Berlin et al., 2014; Hauber et al., 2016; Zhou et al., 2018).
