# Supplementary material for: What Influences Educators’ Design Preferences for Bullying Prevention Programs? Multi-level Latent Class Analysis of a Discrete Choice Experiment
Source: School Ment Health. 2019 Jun 22;12(1):22–37. doi: 10.1007/s12310-019-09334-0 (PMC7021664; doi:10.1007/s12310-019-09334-0)
Supplement: Supplementary file 3 — Supplementary material 3 (DOCX 25 kb) [file 12310_2019_9334_MOESM3_ESM.docx]

| Supplementary Electronic Table 3  *Probability of Membership in Level 2 Educator Classes by Classes of Schools at Level 3* | | | | |
| --- | --- | --- | --- | --- |
|  | Classes of Schools | | | |
|  | Class 1 | | Class 2 | |
|  | *P* | *SE* | *P* | *SE* |
| School Class Size (Level 3) | 0.61 | 0.074 | 0.39 | 0.074 |
| **Classes of Educators (Level 2)** |  |  |  |  |
| All-in Supervisors | 0.22 | 0.026 | 0.28 | 0.035 |
| Facilitators | 0.52 | 0.033 | 0.69 | 0.036 |
| Reluctant Delegators | 0.26 | 0.031 | 0.03 | 0.020 |
|  |  |  |  |  |

*Note.* *P* = probability of membership.
