# Supplementary material for: What Influences Educators’ Design Preferences for Bullying Prevention Programs? Multi-level Latent Class Analysis of a Discrete Choice Experiment
Source: School Ment Health. 2019 Jun 22;12(1):22–37. doi: 10.1007/s12310-019-09334-0 (PMC7021664; doi:10.1007/s12310-019-09334-0)
Supplement: Supplementary file 4 — Supplementary material 4 (DOCX 27 kb) [file 12310_2019_9334_MOESM4_ESM.docx]

Supplementary Electronic Table 4

|  | Classes of Schools | | | |  |  |  |  |
| --- | --- | --- | --- | --- | --- | --- | --- | --- |
|  | Class 1 | | Class 2 | |  |  |  |  |
| Variable | *M* | SD | *M* | SD | F | *p* | η^2^ |  |
| **Theory of Planned Behavior** |  |  |  |  |  |  |  |  |
| Attitudes | 19.31 | 3.95 | 19.88 | 4.07 | 5.01 | .025 | .005 |  |
| Subjective Norms | 21.99 | 4.41 | 23.00 | 4.69 | 12.07 | .001 | .011 |  |
| Perceived Behavioral Control | 16.16 | 4.00 | 17.07 | 3.95 | 12.75 | <0.001 | .012 |  |
| Barriers | 18.57 | 3.94 | 18.17 | 4.42 | 2.26 | .133 | .002 |  |
| Behavioral Intention | 19.37 | 4.47 | 19.77 | 4.68 | 1.84 | .176 | .002 |  |
| **Psychological Reactance** |  |  |  |  |  |  |  |  |
| Psychological Reactance | 13.75 | 6.04 | 12.98 | 5.95 | 4.00 | .046 | .004 |  |
| Dispositional Reactance | 35.77 | 8.25 | 34.43 | 8.99 | 6.04 | .014 | .006 |  |
|  |  |  |  |  |  |  |  |  |

*Theory of Planned Behavior and Psychological Reactance of Educators in Level 3 Latent Classes of Schools*

*Note.* η^2^ = Partial eta squared
